# Long-term survival in patients with non-small cell lung cancer and synchronous brain metastasis treated with whole-brain radiotherapy and thoracic chemoradiation

**DOI:** 10.1186/1748-717X-6-166

**Published:** 2011-11-25

**Authors:** Oscar Arrieta, Cynthia Villarreal-Garza, Jesús Zamora, Mónika Blake-Cerda, María D de la Mata, Diego G Zavala, Saé Muñiz-Hernández, Jaime de la Garza

**Affiliations:** 1 Clinic of Thoracic Oncology, Instituto Nacional de Cancerología (INCan), Mexico City, Mexico; 2 Experimental Oncology Laboratory, INCan, Mexico City, Mexico; 3 Facultad de Medicina, Universidad Nacional Autónoma de México (UNAM), Mexico City, Mexico; 4 Radio-Oncology Department, INCan, Mexico City, Mexico

**Keywords:** NSCLC, brain metastases, chemoradiotherapy, survival

## Abstract

**Background:**

Brain metastases occur in 30-50% of Non-small cell lung cancer (NSCLC) patients and confer a worse prognosis and quality of life. These patients are usually treated with Whole-brain radiotherapy (WBRT) followed by systemic therapy. Few studies have evaluated the role of chemoradiotherapy to the primary tumor after WBRT as definitive treatment in the management of these patients.

**Methods:**

We reviewed the outcome of 30 patients with primary NSCLC and brain metastasis at diagnosis without evidence of other metastatic sites. Patients were treated with WBRT and after induction chemotherapy with paclitaxel and cisplatin for two cycles. In the absence of progression, concurrent chemoradiotherapy for the primary tumor with weekly paclitaxel and carboplatin was indicated, with a total effective dose of 60 Gy. If disease progression was ruled out, four chemotherapy cycles followed.

**Results:**

Median Progression-free survival (PFS) and Overall survival (OS) were 8.43 ± 1.5 and 31.8 ± 15.8 months, respectively. PFS was 39.5% at 1 year and 24.7% at 2 years. The 1- and 2-year OS rates were 71.1 and 60.2%, respectively. Three-year OS was significantly superior for patients with N0-N1 stage disease vs. N2-N3 (60 vs. 24%, respectively; Response rate [RR], 0.03; *p*= 0.038).

**Conclusions:**

Patients with NSCLC and brain metastasis might benefit from treatment with WBRT and concurrent thoracic chemoradiotherapy. The subgroup of N0-N1 patients appears to achieve the greatest benefit. The result of this study warrants a prospective trial to confirm the benefit of this treatment.

## Introduction

Brain metastases occur in 30-50% of patients with Non-small-cell lung cancer (NSCLC) and confer upon the patient a worse prognosis and quality of life [1-6]. Median survival of patients who receive supportive care and are treated only with corticosteroids is approximately 1-2 months [2]. Primary approaches to the treatment of brain metastases include Whole-brain radiation therapy (WBRT), surgery, stereotactic radiosurgery, or a combination, which have achieved a median survival time that ranges from 6.5-10 months [7-11].

As improvements are made in the management of brain metastases, the question arises on how to manage patients with NSCLC who have solely stable brain metastatic disease and on whether treatment should be considered for the primary lung lesion. Long-term survival has been achieved in some patients who have undergone either cranial surgery or radiotherapy and aggressive thoracic management with lung tumor resection, with studies reporting 5-year survival rates between 10 and 20% [12-16]. Few studies have evaluated the role of thoracic radiation or chemoradiotherapy as definitive treatment in the management of patients with NCSLC and synchronous solitary brain metastasis, and some of these have shown promising results [17,18]. Despite these findings, the majority of patients are only offered chemotherapy or radiation therapy in a palliative manner [18].

In an effort to clarify such conflicting data and in order to identify patients who may benefit from aggressive management, we reviewed the outcome of 30 patients with either unresectable single or multiple brain metastases treated with WBRT, who were managed subsequently with definitive thoracic chemoradiotherapy.

## Methods

In a retrospective review of patients treated at the Instituto Nacional de Cancerología (INCan) in Mexico City from May 2005 to March 2009, we identified 30 patients with histologically proven NSCLC and synchronous brain metastases. All patients selected for this analysis had the following characteristics: (1) synchronous diagnosis of NSCLC and brain metastasis (within 2 months of the lung primary diagnosis); (2) absence of neoplastic spread elsewhere in the body at the time of NSCLC and brain metastases detection, and (3) patients with either one unresectable lesion or multiple brain metastases, who were not candidates for surgery or stereotactic radiosurgery treated with WBRT.

Patients' hospital records and office charts were reviewed. Variables collected for analysis included age, gender, Karnofsky performance status (KPS), Radiation Therapy Oncology Group Recursive partitioning analysis (RPA) class, primary Tumor-node-metastasis staging (TNM, according to the American Joint Committee on Cancer Staging Manual, sixth edition [19]), primary histology, and number of brain metastases.

Diagnosis of brain metastases was made based on brain imaging using either Computed tomography (CT) or Magnetic resonance imaging (MRI). These studies were obtained as a routine staging procedure or in the evaluation of suspicious symptomatology. Thoracic stage and nodal status were determined by means of chest Computed tomography (CT) with or without Positron emission tomography (PET) imaging.

### Treatment plan

#### Whole-brain radiotherapy

Conventional megavoltage external beam radiotherapy was administered with a linear accelerator (energy 6 MV) or with a cobalt bomb (1.25 MV). For WBRT, two lateral opposed fields were used, covering up to C2 vertebral body. Dose administered was 30 Gy/10 fractions. Once patients received WBRT, systemic chemotherapy was started.

#### Systemic and thoracic treatment

Initially, two cycles of systemic chemotherapy were administered with paclitaxel 175 mg/m^2 ^Intravenously (IV) > 3 h and cisplatin 75 mg/m^2 ^IV on day 1 every 21 days. Following response assessment after the first two cycles of chemotherapy and in the absence of progression either at the primary lung tumor or Central nervous system (CNS), chemoradiotherapy was indicated.

Thoracic radiotherapy was administered with a linear accelerator (energy 6 and/or 15 MV), and the treatment volume for the primary tumor was based on initial diagnosis volume. The ipsilateral hilum was treated for N0-N2 disease, while for N3, the contralateral hilum was also included. Clinical target volume (CTV) included gross tumor volume plus 2-cm margin and the dose to the primary tumor was 60 Gy (BED).

Concurrently with radiotherapy, weekly paclitaxel 60-80 mg/m^2 ^and carboplatin at a dose of AUC 2, according to Calvert, were administered. If disease progression was ruled out, four chemotherapy cycles followed, using the same schedule as that of the induction regimen.

#### Response assessment

Follow-up for primary disease was performed with CT, generally every 2 months. Follow-up for brain metastases was conducted by MRI, usually every 2 months. All patients were evaluated according to Response Evaluation Criteria in Solid Tumors (RECIST) criteria every two cycles: Complete response was defined as resolution of all disease, partial response constituted a 30% decrease in the sum of the longest diameter of target lesions, progressive disease was defined as 20% increase in the sum of the longest diameter of target lesions, and stable disease comprised neither sufficient shrinkage to qualify for partial response nor sufficient increase to qualify for progressive disease. Response assessment was performed before chemoradiotherapy was indicated.

#### Statistical analysis

For descriptive purposes, continuous variables were summarized as arithmetic means and Standard deviation (SD), and categorical variables as relative frequencies and proportions. Progression-free survival (PFS) was calculated from date of diagnosis of NSCLS until progression. Overall survival (OS) was defined as time from diagnosis of NSCLS until death or until the patient was censored at time of last follow-up. Median time to progression, median probability of survival, and 1- and 2-year survival rates were estimated by the Kaplan-Meier method. Significance value was set at *p *< 0.05. SPSS software package version 17 (SPSS, Inc., Chicago, IL, USA) was employed to analyze the data.

## Results

### Patient Characteristics

Patient characteristics are summarized in Table 1. Median patient age at time of diagnosis was 57 years, and 56.7% of patients were females. Smoking history was documented in 76.7% of patients. The majority of patients had an Eastern Cooperative of Gynecologists (ECOG) performance status of 0 or 1 (90%). The most common histology was adenocarcinoma (80%), and all patients were RPA 2 class. Twenty patients (66.7%) were evaluated with PET CT to rule out other sites of metastatic disease. The number of brain metastatic lesions varied between 1 and 5, with a median of three CNS metastases.

**Table 1:** Baseline characteristics of patients and disease

**Median age ***(years) *	57 ± 11.1
**Gender ***(Female)*	17 (56.7%)
**ECOG**	
0	8 (26.7%)
1	19 (63.3%)
2	3 (10%)

**Comorbidities**	
EPOC	4 (13.3%)
Diabetes	3 (10%)
Hypertension	3 (10%)

**Histology**	
Adenocarcinoma	24 (80%)
Squamous	4 (13.3%)
Other	2 (6.7%)

**Smoking history**	
Yes	23 (76.7%)
No	7 (23.3%)

**Nodal status**	
N 0-1 (n)	16 (53.3%)
N 2-3 (n)	14 (46.7%)

**RPA class 2**	30 (100%)

**Median brain metastatic lesions**	3 ± 2

ECOG: Eastern Cooperative of Gynecologists; RPA: Radiation Therapy Oncology Group Recursive partitioning analysis (RPA).

### Response

With regard to response after WBRT, all patients received the planned radiotherapy dose. Twelve patients (40%) had stable disease, nine patients (30%) achieved partial response, and nine (30%) presented complete response. Median number of chemotherapy cycles was six. Five patients did not receive the planned cycles (17%). When primary tumor response was assessed, 18 patients (60%) achieved a partial response and 12 patients (40%) had stable disease.

### Outcome

**Figure 1:**
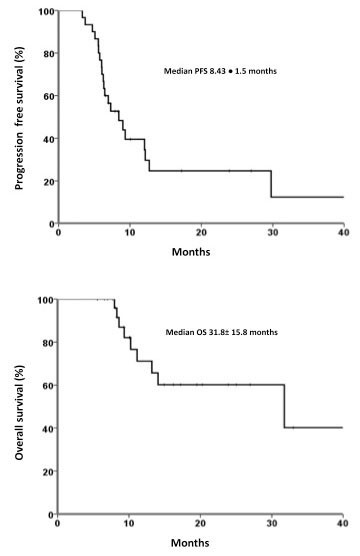
**A) Kaplan-Meier Progression-free survival (PFS) curve for patients with NSCLC treated with WBRT and concurrent chemoradiotherapy (*n *= 30)**. B) Kaplan-Meier Overall survival (OS) curve for patients with NSCLC treated with WBRT and concurrent chemoradiotherapy (*n *= 30).

**Figure 2:**
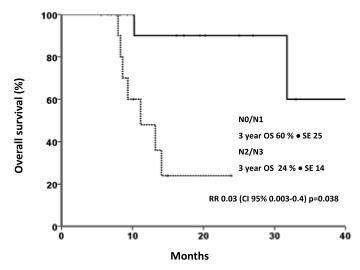
**Overall survival (OS) for patients with NSCLC treated with WBRT and concurrent chemoradiotherapy**.

Median follow-up was 10.2 ± 2 months. Median PFS and OS were 8.43 ± 1.5 months and 31.8 ± 15.8 months, respectively [Figures 1A and 1B]. PFS was 39.5% at 1 year and 24.7% at 2 years. Fifteen patients (50%) had local progression, whereas 10 (33%) developed distant sites of metastases. The 1- and 2-year OS rates were 71.1 and 60.2%, respectively. Three-year OS was significantly superior for patients with N0-N1 stage disease vs. N2-N3 (60 vs. 24%, respectively; RR, 0.03; *p *= 0.038) [Figure 2]. In a univariate analysis using the Log Rank test, there was no statistically significant difference in survival according to age (*p *= 0.07), KPS (*p *= 0.5), global response (*p *= 0.7), history of smoking (*p *= 0.4), and histology (*p *= 0.17).

## Discussion

A considerable proportion of patients with NSCLC develop brain metastases at some point during their disease course and this generally leads to a poor prognosis. However, in some cases, long-term survival has been achieved after aggressive therapy of cerebral lesions, with a median survival of 10-12 months and 5-year survival rates ranging from 10-25% [7,8,20,21].

As improvements are made in the management of patients with brain metastases without extracranial disease, the question arises of how to treat the primary lung cancer. This is due to the fact that the main cause of death in patients with lung carcinoma treated aggressively for brain metastases is progression of the primary cancer [13,22].

In a recent review of the literature, Modi et al. identified 11 papers that addressed the issue of surgical resection of the primary tumor within the context of metastatic brain lesions [23]. They found that median survival for curative intent groups (combined therapy with or without adjuvant treatment) ranged from 19-27 months (mean, 23.12 ± 3.3 months) and 1-, 2-, and 5-year OS reached 56-69%, 28-54%, and 11-24%, respectively. The authors concluded that in the absence of mediastinal lymph node involvement, surgical resection of NSCLC with complete resection of the brain metastasis improves prognosis. Furthermore, they described that some features such as adenocarcinoma histology, low carcinoembryonic antigen (CEA) levels at presentation, response to preoperative chemotherapy before local treatment, and high KPS score may have a positive prognostic value.

The benefit of surgical resection after induction therapy compared with definitive radiotherapy remains uncertain and appears to be associated with an increase in early surgery-associated mortality. The Intergroup 0139 [24] and the EORTC 08941 [25] did not confirm a significant survival advantage for surgery following either neoadjuvant concurrent chemoradiotherapy or induction chemotherapy. Thus, we opted to treat our patients with NSCLC with brain metastases with definitive chemoradiotherapy as an alternative to a more aggressive surgical approach.

To our knowledge, five different studies have reported their results in patients with NSCLC with synchronous brain metastatic disease treated with radiotherapy [13,16-18,26] and/or chemoradiotherapy [17,18] to the primary tumor, but the majority of these studies also included patients treated with other modalities, such as surgical resection of the thoracic lesion [13,16,18] or solely chemotherapy [17]. Brain management differed significantly among these series, and included surgical resection of brain metastases, WBRT, stereotactic surgery, or a combination of the former. Median OS times varied between 5.2 and 18 months, with 1- and 2-year survival rates that ranged between 22-71.3% and 10-34.1%, respectively. For patients treated with definitive thoracic therapy after brain metastases management with gammaknife stereotactic radiosurgery, Flannery et al. reported significantly better survival than those that did not receive definitive treatment (median OS, 26.4 vs. 13.1 months). In our series, we reached a median survival of 31.8 months, the longest survival reported to date in patients with synchronous brain metastatic disease treated with definitive chemoradiation to the primary. Unlike other studies, in our report, treatment delivered to the brain, as well as systemic and local therapy, was homogeneous among all patients included.

By applying American Joint Committee on Cancer (AJCC) staging only to the primary site, Hu et al. reported that patients with thoracic stage I had a more favorable outcome, with a median survival time of 25.6 months (compared with 9.5 and 9.9 months for stages II and III, respectively), and concluded that aggressive treatment to the lung may be justified for newly diagnosed thoracic stage I NSCLC with a solitary brain metastasis [17]. Also, Louie et al. reported longer overall survival for stage I/II disease of 14.7 months compared with 7 months for patients with stage III NSCLC with synchronous solitary brain metastasis treated with craniotomy and WBRT [16]. In our study, according to the univariate analysis, we found that patients with N0-N1 disease had a significantly better 3-year survival rate compared with those with N2-N3 disease. Similar findings have been reported in previous studies where the absence of mediastinal lymph node involvement (N2) is one of the most important survival determinants [15,27,28]. Accordingly, in patients with controlled brain disease and early lung disease (N0-N1) amenable to resection, a conservative surgery of the primary tumor may be the best treatment alternative (segmentectomy or lobectomy). For patients who are not candidate for surgical resection, chemoradiatiation to the primary tumor should be considered. In Table 2, we condensed the studies that assessed radiation therapy or chemoradiotherapy of the primary lung tumor in patients with central nervous metastatic disease.

**Table 2:** Series evaluating radiation therapy or chemoradiotherapy of lung cancer in NSCLC with brain metastasis

Study	Year	Number of patients	Number of brain metastases	Brain treatment	Median survival, months	1-year survival rate (%)	2-year survival rate (%)	5-year survival rate (%)	Prognostic factors
Chidel et al.[13]	1999	Sx = 2 Sx + RT = 3 RT = 8 Palliative = 15	1	Sx WBRT STS and combination	Overall: 6.9 Definitive tx: 20.1 No definitive tx: 3.5		12 (3-year)		WBRT Aggressive thoracic treatment

Moazami et al.[31]	2002	Chemo/RT = 29 Sx = 59	42 pts: 1 49 pts: > 1 Metachronous brain lesions	Sx WBRT STS and combinations	5.2	22	10	-	Younger age Stage IIIA Lung resection ECOG No extracranial mets Sx of brain mets STS

Hu et al.[17]	2006	RT = 44 Chemo = 23 Chemo/RT = 13	1	Sx STS	Overall: ~15 Stage I: 25.6 Stage II: 9.5 Stage III: 9.9	49.8	16.3	7.6	Thoracic stage I

Ampil et al.[26]	2007	> 65 year RT = 22 No RT = 50	1	WBRT STS Sx and combinations	RT = 5 No RT = 3 *p *= 0.28	RT = 14 No RT = 11 *p*= 0.28	-	-	Sx of brain mets

Flannery et al.[18]	2008	RT = 26 Chemo/RT = 9 Sx = 12 Preop Chemo + Sx = 5	1	STS	Overall: 18 Definitive tx: 26.4 No definitive tx: 13.1 *p *< 0.0001	Overall: 71.3	Overall: 34.1	Overall: 21 RT: 34.6 No RT: 0 *p *< 0.0001	Definitive thoracic therapy KPS

Louie et al.[16]	2009	Sx = 8 Chemo = 24 RT = 14	1	Sx + WBRT	7.8 Stage I/II: 14.7 Stage III: 7	-	-	-	Lung surgery^a^ Primary lung treatment^a^ > 8 weeks after brain Sx^a^ Stage I/II disease^a^

Arrieta et al. (present series)	2010	Chemo-Chemo/RT = 30	> 1	WBRT	31.8	71.1	60.2		3-year OS for N0-1 was 60 vs. 24% for N2-3

NSCLC: Non-small-cell lung cancer; Sx: Surgery; RT: Radiotherapy; WBRT: Whole-brain radiotherapy; STS: Stereotactic surgery; Comb: Combination; Mets: Metastasis/Metastases; Chemo: Chemotherapy; Chemo/RT: Chemo-radiotherapy; Preop: Pre-operative. a) Univariate analysis.

To our knowledge, this is the first time that patients with multiple synchronous brain metastases treated with WBRT have been managed with aggressive therapy for primary tumor in chest with concurrent chemoradiotherapy, showing a very long OS. Among series that evaluated surgery as local treatment for the thoracic disease, several included patients with multiple brain metastases [12,28-32]. In none of these studies was the number of brain lesions described as a factor that conferred poor prognosis.

Other prognostic factors associated with better outcomes with this definitive strategy after treatment to the brain disease are younger age [31,33], early stage nodal disease [15-17,27,28,31], good performance status [18,30-32,34], adenocarcinoma subtype [14,28,32,35], location of primary tumor, site of brain metastases, and low CEA levels [28,36].

Median survival in patients treated in this series is remarkably long. One of the reasons might be that we selected patients who presented with synchronous brain tumors and did not include those with metachronous lesions, which may represent a better prognostic group. In addition, we selected patients who did not progress after induction chemotherapy to proceed with further thoracic management, in order to ensure that patients with rapid progressive metastatic disease would not be submitted to a more aggressive management instead of a palliative approach. Furthermore, the majority of patients were assessed with PET-CT at diagnosis, which reduces the likelihood of metastatic disease elsewhere, compared with routine evaluation with CT and bone scan [37]. Finally, a high proportion of patients had stage 0-1 nodal disease (53.3%) and 0-1 ECOG grade (90%) and with few comorbidities, which might suggest that this therapeutic approach should be reserved for otherwise healthy patients with non-bulky mediastinal lymph node involvement.

## Conclusion

Patients with NSCLC and synchronous brain metastases might benefit from aggressive treatment with WBRT and concurrent chemoradiotherapy to the primary lesion, particularly in well-selected patients. The result of this study warrants a prospective trial in stage IV disease with only brain metastasis to confirm the benefit of this treatment.

## Conflict of interests

The authors declare that they have no competing interests.

## Authors' contributions

OA conceived of the study, and participated in its design and coordination. CV participated in its design and drafted the manuscript. JZ performed the patient selection and analysis. MB performed the patient selection and analysis. MDM participated in patient selection, analysis and manuscript draft. DGZ participated in the manuscript draft. SM performed the statistical analysis. JG participated in its design and coordination. All authors read and approved the final manuscript.
